# Research on breastfeeding: Α call to action

**DOI:** 10.18332/ejm/117953

**Published:** 2020-03-09

**Authors:** Asimenia Mamakou, Chrysanthi Chavredaki

**Affiliations:** 1 General, Obstetrics and Pediatric Clinic Mitera, Athens, Greece; 2 Department of Labour Ward and Obstetrics and Gynaecology Clinic, General Hospital of Chania, Chania, Greece

**Keywords:** breastfeeding, economic benefits, economic impact, breastfeeding benefits

Social science and medical research show that experiences in childhood and early childhood, including health and cognitive development, have lasting effects in life – one such positive experience is breastfeeding. Breast milk contains: lipids, vitamins, sugars, proteins, carbohydrates, antibodies, and bioactive molecules. It promotes nourishment and growth of the infant, and provides protection against inflammation and infections. Its composition is not stable but depends on genetic, environmental and nutritional factors during the lactation period, but also on the birth of the newborn.

Breastfeeding infants have multiple health benefits, such as lower incidence of gastrointestinal, respiratory and otitis infections as well as a reduced risk for acute lymphoblastic leukaemia and sudden death syndrome^1^. The findings of a 2015 meta-analysis show a correlation of maternal breastfeeding duration with an increase in the person's intelligence index^2^. In another study by Hanushek and Wössmann^3^, the index of intelligence was correlated with the hourly earnings in both high-income and low-income countries, indicating indirect benefit.

The incidence of reduced morbidity in healthcare costs was estimated by the cost of common neonatal and childhood conditions in four countries (USA, UK, China, and Brazil) based on the incidence of breastfeeding, indicating the significant effect of breastfeeding on the economy of each country^4^.

For every 10% increase in exclusive breastfeeding beyond six months, or the continuation of breastfeeding for one or two years, translates into a reduction in the cost of treatment of childhood illnesses, at least by $312 million in the US^5,6^.

Interventions aimed at creating a supportive environment for breastfeeding in primary care are, therefore, necessary. These include legislative initiatives, strengthening the role of health professionals in breastfeeding issues in primary health care, and initiatives such as the Baby-Friendly Hospital Initiative.

Breastfeeding is an essential aspect of maternal and neonatal health identified across the globe, with research published in the European Journal of Midwifery underlining the common necessity to support breastfeeding initiatives in a plethora of countries such as Ethiopia^7^, Greece^8^ and Norway^9^.

Breastfeeding contributes to a world that is healthier, better educated, fairer and more environmentally sustainable. Let us, through the European Journal of Midwifery, make this evident.
